# Impact of Neuroimaging Patterns for the Detection of Atrial Fibrillation by Implantable Loop Recorders in Patients With Embolic Stroke of Undetermined Source

**DOI:** 10.3389/fneur.2022.905998

**Published:** 2022-06-13

**Authors:** Joong-Goo Kim, Kiyung Boo, Chul-Hoo Kang, Hong Jun Kim, Jay Chol Choi

**Affiliations:** ^1^Department of Neurology, Jeju National University Hospital, Jeju, South Korea; ^2^Department of Internal Medicine, Jeju National University Hospital, Jeju, South Korea

**Keywords:** atrial fibrillation, cerebral infarction, embolic stroke of undetermined source (ESUS), implantable loop recorder (ILR), neuroimaging

## Abstract

**Objectives:**

Atrial fibrillation (AF) is a well-known etiology of embolic stroke of undetermined source (ESUS), although the optimal detection strategy of AF was not been fully evaluated yet. We assessed AF detection rate by implantable loop recorder (ILR) in patients with ESUS and compared the clinical characteristics and neuroimaging patterns between the patients with AF and AF-free patients.

**Methods:**

We reviewed clinical characteristics and neuroimaging patterns of consecutive patients with who were admitted to our comprehensive stroke center for ESUS and underwent ILR insertion between August 1, 2019, and January 31, 202. The inclusion criteria were (1) 18 years of age or older; (2) classified as having cryptogenic stroke extracted from the group with undetermined stroke according to ESUS International Working Group; and (3) underwent ILR insertion during or after admission due to index ischemic events. Ischemic stroke pattern was classified as (1) tiny-scattered infarction, (2) whole-territorial infarction, (3) lobar infarction and (4) multiple-territorial infarction. Interrogations of data retrieved from the ILR were performed by cardiologists in every month after the implantation.

**Results:**

In this study, 41 ESUS patients who received an ILR implantation were enrolled (mean age, 64 years; male sex, 65.9%). The rate of AF detection at 6 months was 34% (14 patients), and the mean time from ILR insertion to AF detection was 52.5 days [interquartile range (IQR), 45.0–69.5]. The median initial NIH stroke scale scores were significantly greater in patients with AF than those without AF (6.5 vs. 3.0, *p* = 0.019). Whole-territorial infarction pattern was significantly more frequent in patients with AF than in those without AF (64.3% vs.11.1%, *p* = 0.002).

**Conclusions:**

Higher covert AF detection rates within the ESUS patients were most often associated with higher NIHSS and whole-territorial infarction patterns on brain imaging.

## Introduction

The clinical spectrum of embolic stroke of undetermined source (ESUS) is defined as patients with ischemic stroke for whom neither a cardioembolic nor a non-cardiac source can be detected during the initial evaluation (1). ESUS is a common stroke subtype that accounts for 23–40% of all strokes (2). Covert atrial fibrillation (AF) is a predominant etiology of ESUS, although the risk of embolism can be prevented by anticoagulation (3). Thus, cardiac monitoring remains the cornerstone in evaluating ESUS patients with suspected AF and the prevention of recurrent stroke (4). Strategies for the detection of covert AF in ESUS have included in-hospital monitoring, serial electrocardiography (ECG), Holter monitoring, monitoring with the use of external events, long-term outpatient monitoring, and monitoring by implantable loop recorders (ILR), but the detection rates range from 0 to 25%, indicating low effectiveness (1, 5) because most covert AF is often asymptomatic and paroxysmal, symptom-driven monitoring or intermittent short-term recordings seem less effective, and the diagnostic yield might depend on the monitoring duration (6). Recent studies suggested that AF was more frequently detected by ILR than by conventional monitoring methods in patients with recent ESUS (3). Data from long-term cardiac monitoring by ILR could provide an optimal strategy for quantifying the likelihood of detection by less complicated strategies. However, the cause of embolism in ESUS patients is arrhythmias such as AF, along with various other causes such as aortic arch atheroma, patent foramen ovale, and malignancy (1, 7). For patients included in the ESUS category, the classification of patients in whom ILR can most effectively detect asymptomatic AF is under investigated at present. Thus, among patients with ESUS who received ILR screening, we sought to evaluate the efficacy of the real practical achievements of ILR strategies in the detection of subclinical AF and assess neuroimaging patterns and clinical characteristics to determine whether AF is associated with the diagnostic yield.

## Methods

### Study Population

This retrospective study was performed at a single comprehensive stroke center in South Korea between 1 August 2019, and 31 January 2021. According to our management protocol, ILR insertion was considered for all the patients with ESUS during the study period. However, ILR implantation was not considered for patients who did not undergo the full evaluation for the diagnosis of covert embolism or who potentially had multiple causes of systemic embolization upon admission to the hospital. ILR insertion was also not performed when patients or their proxy did not consent or when a medical condition contraindicated ILR insertion. In this study, we included consecutive ESUS patients who (1) were 18 years of age or older; (2) were classified as having cryptogenic stroke extracted from the group with undetermined stroke according to ESUS International Working Group (2); and (3) underwent ILR insertion during or after admission due to index ischemic events. The main exclusion criteria were patients with a history of AF or valvular heart disease or patients who required permanent anticoagulation therapy due to any underlying hypercoagulable disease. Additionally, we excluded patients (1) with a persistent neurological deficit that was potentially severe disabling; (2) when the observation period after ILR insertion was <180 days; and (3) in cases with AF detected immediately before or after ILR insertion (within 7 days). Patients with two or more potential embolic sources demonstrated during the initial evaluation were not excluded. This study was approved by the institutional review board of our hospital, and the need for written informed consent was waived because of the retrospective nature of the study.

### ESUS Evaluation Protocol

Included patients had received a diagnosis of acute ischemic stroke or transient ischemic attack (TIA) occurring within the previous 7 days according to brain magnetic resonance imaging. Stroke was classified as ESUS, if extensive evaluation failed to reveal a definite embolic origin, such as continuous stroke unit (or intensive care unit) electrocardiogram (ECG) monitoring, daily 12-lead ECG monitoring for at least 3 days, and 24-h ECG monitoring (Holter). Additionally, screening for hypercoagulable states (in patients <60 years of age) and neuroimaging, such as magnetic resonance angiography (MRA), computed tomography angiography (CTA), or digital subtraction angiography of the head and neck, were performed upon initial admission. Extracranial carotid duplex ultrasonography and transcranial Doppler ultrasonography were performed for all patients.

### Neuroimaging Patterns

**Figure 1:**
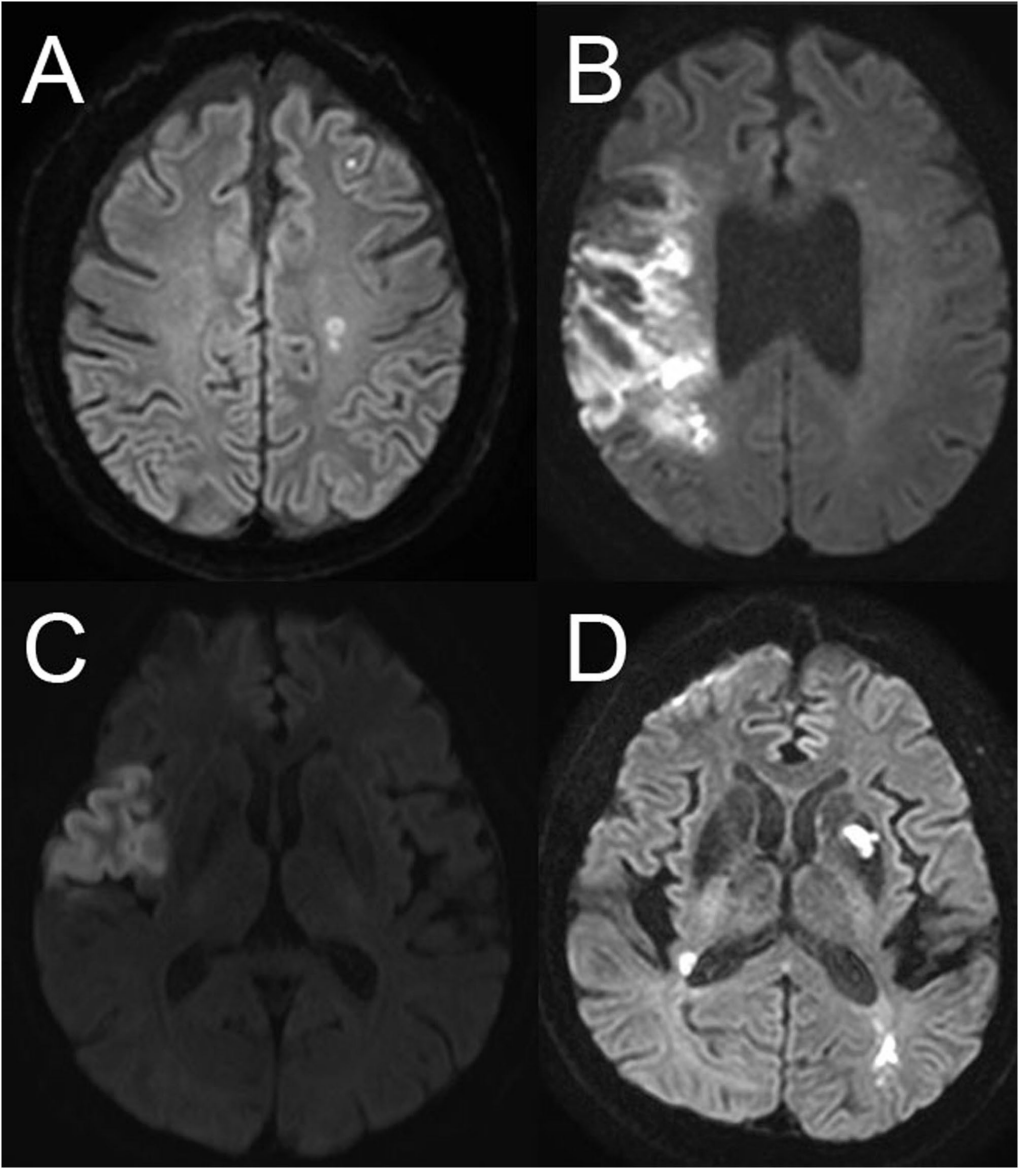
Neuroimaging patterns of embolic stroke of undetermined source. **(A)** Scattered infarctions, **(B)** whole-territorial infarction, **(C)** lobar infarction, **(D)** multiple-territorial infarction.

Embolic stroke patterns were evaluated retrospectively using CTA, MRA, or digital subtraction angiography performed at the time of admission or during the in-hospital stay following the index ischemic stroke. Neuroimaging was evaluated if the patients were classified as having ESUS after an extensive initial workup. Patterns of ESUS were analyzed in four groups: tiny-scattered infarctions; whole-territorial infarction; lobar infarction; and multiple-territorial infarction. The tiny-scattered infarction pattern was defined as multiple non-contiguous lesions, which were hyperintense on diffusion weighted imaging (DWI) and hypointense on apparent diffusion coefficient (ADC) maps within the same vascular territory. Whole-territorial infarction was defined as a whole-territorial infarct with/without large-vessel occlusion or major ischemic symptoms and TIA-like global aphasia with right-side weakness. Large vessels were defined as the internal carotid artery (ICA), first and second segments of the middle cerebral artery, anterior cerebral artery, vertebral artery, basilar artery, and proximal posterior cerebral artery. Lobar infarction was defined as a wedge-shaped infarct in the anterior circulation with/without large branch occlusion. Multiple-territorial infarction was defined on neuroimaging as noncontiguous infarcts located in more than vascular territories (Figure 1). The classification criteria for neuroimaging pattern were devised by referring to previous research on the lesion pattern in patients with ESUS (8, 9). The first author of the study (JG Kim) reviewed all the neuroimaging data and classified the stroke lesion patterns.

### Pathway of Referral to the Electrophysiologist for Fast ILR Implantation

We implemented a pathway of referral to the electrophysiologist for fast ILR implantation on August 1, 2019. Before August 2019, we did not have a written protocol for ILR insertion. Instead, we routinely performed an embolic workup on the scheduled date according to the cardiologist's schedule. After basic cardiac evaluation, such as echocardiography and 24-h Holter monitoring, the general cardiologist was consulted for the insertion of ILR. The decision of ILR insertion was made by the treating stroke neurologists. Since implementing the protocol of the electrophysiologist referral pathway, we routinely screened patients according to the inclusion criteria and exclusion criteria. We consulted the electrophysiology staff directly, and basic cardiac examinations such as echocardiography and 24-h Holter monitoring were performed quickly during the hospitalization period. In addition, during the acute phase, continuous cardiac monitoring was performed in the stroke unit or intensive care unit, and ECG was performed daily for at least 3 days. Through this protocol, our best efforts were made to ensure that patients could receive ILR insertion in the acute stage of ESUS.

### Implant Procedure

The ILR was implanted in a left parasternal position. The optimal orientation of the device was determined in advance using the Vector Check Tool (Medtronic), which enables testing for the highest R-wave amplitude obtained from single-lead recordings in different locations and orientations on the body surface. The device was inserted into the subcutaneous tissue with limited prior pocket preparation to ensure close tissue-device contact. The sensitivity was programmed to 0.035 mV (35 μV), and the delay after sensing was 150 ms. The devices were programmed to detect AF with standard bradycardia and tachycardia detection limits (<40 bpm and >150 bpm).

### ILR Monitoring

Interrogations of data retrieved from the ILR were performed by cardiologists 1–2 months after implantation. The monitoring type and duration and all results were recorded. All patients were scheduled for device insertion within 7 days after the required cardiac evaluation. The settings of inserted ILRs were programmed following a standardized protocol. The ILR (REVEAL LINQ, Medtronic) automatically detects and records AF, irrespective of heart rate or symptoms. All patient follow-up visits were scheduled every 1 month, with unscheduled visits in the event of symptom occurrence. If patients reported an episode of AF after the previous visit, clinical information was provided to the stroke neurologists.

### Clinical Assessment and Data Acquisition

Patient data were retrospectively retrieved from the prospective database of our institute. Factors compared between the two groups were extracted from electronic medical records and included demographic characteristics (e.g., age, sex, and cerebrovascular risk factors); clinical characteristics (e.g., initial ECG, CHA_2_DS_2_-VASc score, National Institutes of Health Stroke Score (NIHSS) scores at baseline and discharge, length of hospital stay and times from index stroke to transthoracic echocardiography (TTE), Holter monitoring, and the detection of AF); and radiographic and angiographic characteristics (e.g., stroke location, neuroimaging pattern, acute stroke treatment, and detailed patient characteristics).

### Statistical Analyses

We compared baseline characteristics, clinical status, and imaging patterns between patients with detected AF and without detected AF during the follow-up periods. For univariable analysis, we used the Pearson chi-square test, Fisher's exact test, and the Mann–Whitney U-test, as appropriate. Multivariate logistic regression analysis was performed to determine the independent contributions of variables to the detection of AF. Variables with a *p*-value <0.2 in the univariate analysis were included as candidate variables in the multivariate analysis and removed by backward stepwise selection. Additional analysis by forward selection confirmed the final model. A two-tailed *p*-value <0.05 was considered to indicate a significant difference in all statistical analyses. All statistical analyses were performed with SPSS version 21.0 (IBM, Armonk, NY, USA).

## Result

**Figure 2:**
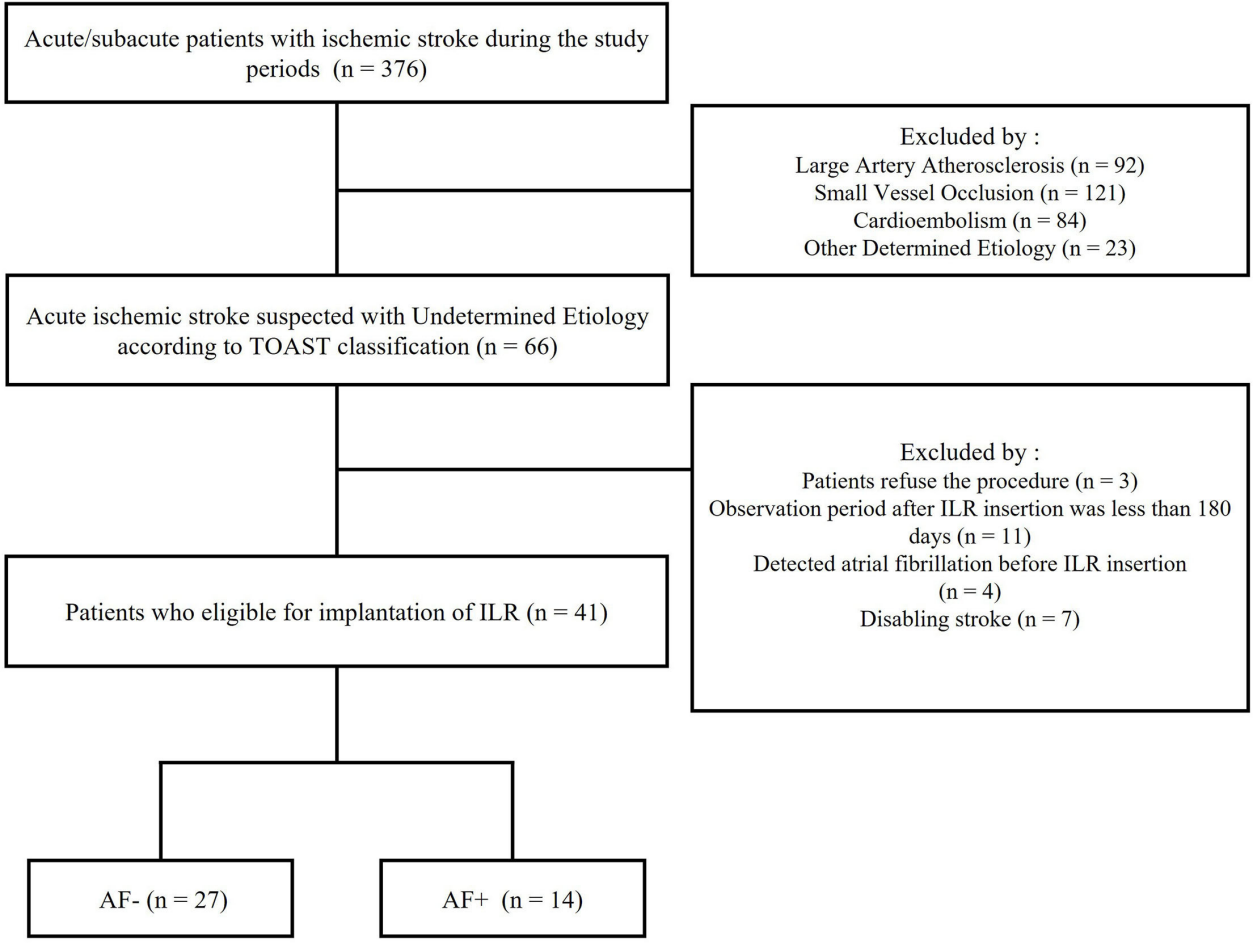
Flowchart of the Patient selection process.

The patient flowchart is shown in Figure 2. All patients with acute ischemic stroke without evidence of an embolic source were classified as having ESUS. A total of 376 acute ischemic stroke patients were admitted to our hospital between 1 August 2019, and 31 January 2021. Of these 376 patients, 310 were excluded, such as 92 with large-vessel atherosclerotic disease, 121 with small-vessel occlusion, 84 with cardioembolism, and 23 with undetermined sources. Finally, 66 patients with acute ischemic stroke who suspected ESUS were included. Based on exclusion criteria, 25 patients were excluded. The rate of AF detection at 6 months was 34% among all study patients (14 patients), and the median time from ILR insertion to AF detection was 52.5 days (interquartile range, 45 to 69.5).

AF- and AF+ patients showed no significant differences in baseline characteristics, such as initial ECG and CHA_2_DS_2_-VASc scores, but hypertension, smoking, and alcohol intake presented small differences between the two groups (Table 1). Although prestroke modified Rankin Scale (mRS) scores did not differ significantly between these groups, the median initial NIHSS scores were significantly more severe in the AF+ group than in the AF- group [three (interquartile range, IQR)1.0–6.0) vs. 6.5 (IQR, 2.0–16.0); *p* = 0.019]. There were no significant differences among patients who received mechanical thrombectomy, TEE or warfarin anticoagulation therapy. The median times from index stroke to TEE [6.0 (IQR, 3.0–18.0) vs. 5.0 (IQR, 2.0–10.0) days; *p* = 0.337] and from index stroke to 24-h Holter monitoring [3.5 (IQR, 2.0–6.0) vs. 3.5 (IQR, 2.0–6.0)) days; *p* = 0.926] were not significantly different between the groups. Additionally, the median times from stroke to AF detection [58.0 (IQR, 49.0–69.5) vs. 52.5 (IQR, 44.5–97.0) days; *p* = 0.671] were not significantly different between the groups. However, the detection of AF with the whole-territorial infarction pattern was significantly higher in AF+ patients than in AF- patients [AF+: 9 (64.3%) vs. AF-:3 (11.1%)]. However, multiple-territory infarction patterns were much more frequent in the AF- group than in the AF+ group [AF-: 8 (29.6%) vs. AF+: 2 (14.3%); *p* = 0.002]. The middle cerebral artery was more commonly the involved vascular territory in AF+ patients than in AF- patients [AF-: 9 (33.3%) vs. AF+: 12 (85.7%); *p* = 0.004] (Table 2). The median NIHSS scores at discharge [1.0 (IQR, 0.0–3.0) vs. 1.5 (IQR, 0.0–3.0); *p* = 0.746] were similar between the groups, and the discharge mRS scores were not significantly different between the groups (Table 3).

**Table 1:** Baseline characteristics.

	**Total**	**AF-**	**AF+**	***P***
	**(*n* = 41)**	**(*n* = 27)**	**(*n* = 14)**	
Age, years	64.0 (54.0–75.0)	66.0 (54.0–75.0)	64.0 (59.0–73.0)	0.804
Sex, male sex	27 (65.9%)	16 (59.3%)	10 (71.4%)	0.671
Height, cm	166.0 (157.0–172.2)	163.2 (155.2–172.6)	167.7 (164.0–170.0)	0.425
Weight, kg	68.0 (57.5–74.9)	68.7 (57.5–75.9)	66.2 (60.0–73.1)	0.837
Baseline mRS				0.574
0	33 (80.5%)	21 (77.8%)	12 (85.7%)	
1	6 (14.6%)	5 (18.5%)	1 (7.1%)	
2	2 (4.9%)	1 (3.7%)	1 (7.1%)	
Initial ECG				0.539
Normal sinus rhythm	32 (78.0%)	21 (77.8%)	11 (78.6%)	
Conduction block	1 (2.4%)	0 (0.0%)	1 (7.1%)	
Sinus tachycardia	2 (4.9%)	1 (3.7%)	1 (7.1%)	
Sinus bradycardia	5 (12.2%)	4 (14.8%)	1 (7.1%)	
Premature atrial complex	1 (2.4%)	1 (3.7%)	0 (0.0%)	
Comorbidities and risk factors				
Hypertension	27 (65.9%)	21 (77.8%)	6 (42.9%)	0.059
Diabetes mellitus	11 (26.8%)	10 (37.0%)	1 (7.1%)	0.094
Hypercholesterolemia	20 (48.8%)	14 (51.9%)	6 (42.9%)	0.828
Coronary artery disease	4 (9.8%)	3 (11.1%)	1 (7.1%)	1
Smoking	13 (31.7%)	8 (29.6%)	5 (35.7%)	0.966
Alcohol	16 (39.0%)	9 (33.3%)	7 (50.0%)	0.484
Active cancer	0 (0.0%)			
Previous stroke	7 (17.1%)	5 (18.5%)	2 (14.3%)	1
Patent foramen ovale (with R-L shunt)	9 (22.0%)	5 (18.5%)	4 (28.6%)	0.734
CHA_2_DS_2_-VASc score				0.367
0	10 (24.4%)	4 (14.8%)	6 (42.9%)	
1	6 (14.6%)	4 (14.8%)	2 (14.3%)	
2	7 (17.1%)	4 (14.8%)	3 (21.4%)	
3	7 (17.1%)	5 (18.5%)	2 (14.3%)	
4	2 (4.9%)	2 (7.4%)	0 (0.0%)	
5	8 (19.5%)	7 (25.9%)	1 (7.1%)	
6	1 (2.4%)	1 (3.7%)	0 (0.0%)	
Initial NIHSS	4.0 (1.0–6.0)	3.0 (1.0–5.0)	6.5 (2.0–16.0)	0.019

*mRS, Modified Rankin Score; ECG, Electrocardiogram; NIHSS, National Institutes of Health Stroke Scale. Data were N (%) or median (interquartile range)*.

**Table 2:** Initial neuroimaging pattern.

	**Total**	**AF-**	**AF+**	***p***
	**(*n* = 41)**	**(*n* = 27)**	**(*n* = 14)**	
Neuroimaging pattern			
Tiny scattered	12 (29.8%)	11 (40.7%)	1 (7.1%)	0.06
Whole-territorial infarction	12 (29.2%)	3 (11.1%)	9 (64.3%)	0.002
Lobar infarction	7 (17.1%)	5 (18.5%)	2 (14.3%)	1
Multiple territory infarction	10 (24.4%)	8 (29.6%)	2 (14.3%)	0.48
Infarcted territory				
Anterior cerebral artery	1 (2.4%)	1 (3.7%)	0 (0.0%)	1
Middle cerebral artery	22 (53.7%)	9 (33.3%)	12 (85.7%)	0.004
Posterior cerebral artery	1 (2.4%)	1 (3.7%)	0 (0.0%)	1
Internal carotid artery	1 (2.4%)	0 (0.0%)	1 (7.1%)	1
Vertebral artery	4 (9.8%)	4 (14.8%)	0 (0.0%)	0.337
Basilar artery	8 (19.5%)	8 (29.6%)	1 (7.1%)	0.211
Multiple territory	4 (9.8%)	4 (14.8%)	0 (0.0%)	0.337

*TIA, transient ischemic attack*.

**Table 3:** Outcomes according to the pattern of ESUS.

	**Total**	**AF-**	**AF+**	***P***
	**(*n* = 41)**	**(*n* = 27)**	**(*n* = 14)**	
Time to transthoracic echocardiography, days	6.0 (2.0–14.0)	6.0 (3.0–18.0)	5.0 (2.0–10.0)	0.337
Time to Holter monitoring, days	3.0 (2.0–6.0)	3.5 (2.0–6.0)	3.5 (2.0–6.0)	0.926
Time to the first detection of atrial fibrillation, days	52.5 (45.0–69.5)	58.0 (49.0–69.5)	52.5 (44.5–97.0)	0.671
Mechanical thrombectomy	8 (19.5%)	3 (11.1%)	5 (35.7%)	0.142
Transesophageal echocardiography	14 (34.1%)	10 (37.0%)	4 (28.6%)	0.846
Warfarin	8 (19.5%)	4 (14.8%)	4 (28.6%)	0.523
Discharge NIHSS	1.0 (0.0–3.0)	1.0 (0.0–3.0)	1.5 (0.0–3.0)	0.746
Length of hospital stay, days	8.0 (6.0–13.0)	8.0 (6.0–12.0)	8.0 (6.0–13.0)	0.814
Discharge mRS				0.105
0	9 (22.0%)	6 (22.2%)	3 (21.4%)	
1	18 (43.9%)	12 (44.4%)	6 (42.9%)	
2	6 (14.6%)	3 (11.1%)	3 (21.4%)	
3	6 (14.6%)	6 (22.2%)	0 (0.0%)	
4	2 (4.9%)	0 (0.0%)	2 (14.3%)	

*ESUS, Embolic stroke of undetermined source; NIHSS, National Institutes of Health Stroke Scale; mRS, modified Rankin Score*.

## Discussion

Clinically sustained AF documented by 12-lead electrocardiogram (ECG) or ECG monitoring is regarded to be a major pathogenesis of embolic infarct in patients with ESUS (10, 11). Additionally, asymptomatic paroxysmal AF revealed during embolic evaluation is believed to contribute to the etiology of embolic stroke in patients with ESUS (3). Oral anticoagulation is known to more effectively prevent ischemic stroke in AF than in other embolic causes (12, 13). In this study, we focused on identifying ESUS patients with a clinically high probability of cardioembolism. Therefore, daily ECG monitoring (in the stroke unit) and 12-lead ECG were performed in addition to basic embolic evaluation at the beginning of hospitalization. Thus, AF was found in 34.1% of all patients with ESUS, and the average time from ILR insertion to AF detection was 52.5 days. Importantly, AF was diagnosed in 64.3% of patients with whole-territorial infarction patterns in initial neuroimaging. The presence of major-vessel occlusion is also a useful predictor of occult AF in the previous report (14, 15). We analyzed more expanded concepts, such as the patients with major territorial infarctions without significant major vessel occlusion or apparently transient ischemic symptoms such as global aphasia with right-side weakness. Also, we thought the isolated major vessel occlusion with scattered pattern infarction has to consider not only embolism but also intracranial atherosclerotic occlusion. Yushan et al. reported that the bilateral infarcts pattern was significantly associated with AF detection (16). Additionally, larger infarct volumes, infarct size, subsequent infarct growth, and hemorrhagic transformation have been associated with cardiac embolism of stroke (15, 17). Thus, neuroimaging patterns like whole territorial infarction are more reasonable predicting factors for AF in patients with ESUS rather than major vessel occlusion alone. In this study, the neuroimaging pattern was further subdivided and analyzed, and the whole territorial infarct pattern was investigated to be highly correlated with AF.

On the other hand, the initial median NIHSS score of 6.5 in AF+ patients is considered a very important result in this study. Compared to the Cryptogenic Stroke and Underlying AF (CRYSTAL AF) trial, which demonstrated that AF was detected at a rate of 8.9% at 6 months (3), the AF detection rate was significantly higher in our study. This high rate is likely related to the characteristics of the patients enrolled in the two studies. NIHSS scores in most studies of ESUS with cardioembolism were reported to be approximately 4–6 (7, 18–20). In another study analyzing imaging patterns according to the causes of embolism within ESUS patients, the AF group showed mostly territorial/lobar infarct patterns, and the NHISS scores were six or higher (18), as observed in our study. However, the mean NIHSS score of patients enrolled in the CRYSTAL AF group was 1.6 ± 2.7 (3), which was similar to those of the aortic arch atheroma and PFO groups, who showed lower NIHSS scores, and these values were much lower than those in our study. This consideration prompted the expansion of the considered causes of failure of randomized clinical trials (RESPECT-ESUS and NAVIGATE-ESUS trials) evaluating the safety and efficacy of rivaroxaban and dabigatran in patients with ESUS (10, 11). According to CRYSTAL AF, at least more than 30% of ESUS patients potentially have AF (3), and anticoagulation therapy has been predicted to have a preventive effect against ischemic stroke in patients with ESUS. However, the results of two prospective clinical trials that failed to support this hypothesis have called into question the study concept and design, such as patient selection (21, 22). Many analyses of the causes of failure in these studies have been conducted, and many clinical studies on ESUS have started to focus on identifying other embolic causes and analyzed the ECGs of patients to identify atrial cardiomyopathy; moreover, studies have been conducted on cardioembolism that had previously been underestimated, such as left atrial enlargement (7). However, considering our study results, the main reason for previous ESUS trial failure seems to be that the enrolled patients were biased toward mild ischemic symptoms and did not truly have AF, rather than an improper concept of ESUS or a lack of anticoagulation effect (23, 24). Based on the concept of ESUS, trials might have included a heterogeneous group of patients with embolic cerebral infarction. There is a high possibility that most of the patients enrolled in both studies did not have cardioembolic infarction because the median NIHSS score of patients enrolled in the RESPECT-ESUS and NAVIGATE-ESUS trials was only one (10, 11). Thus, considering the results of our study, future studies should focus on analyzing patient clinical features, and neuroimaging patterns might provide better information than other complicated embolic sources or advanced study designs. It is important to identify who can benefit the most from ILR insertion, even among ESUS patients. The results from these trials imply that patients with ESUS cannot be treated simply as a unified entity, and more research is needed to identify the optimal therapy for each ESUS patient subgroup.

The clinical characteristics and neuroimaging-based decisions showed a good AF detection rate. Our study showed the clear benefit of ILR insertion for detecting AF in patients with ESUS who had whole-territorial infarction and high initial NIHSS scores. Additionally, these results suggest the need to identify which patients would derive the most clinical benefit from AF detection and anticoagulation therapy among ESUS patients. Although the A-fib detection rate in patients was more than 30% during the ILR monitoring period in our study, regardless of the neuroimaging pattern, it is necessary to consider which ESUS patients should undergo ILR insertion, and the inclusion criteria for anticoagulation therapy for ESUS in future studies should include patients with a high probability of covert AF, rather than all ESUS patients.

This study had several important limitations. This single-center study was retrospective, and the patient selection for ILR insertion depended on the preference of the duty neurologist, which might have resulted in selection bias. However, our baseline data showed a relatively balanced distribution of clinical variables between the groups, which suggested minimal limitations related to the retrospective nature of the study. Additionally, the results of this study should be interpreted with caution because the number of enrolled patients was small, and the duration of the observation period was regarded as relatively insufficient. Nevertheless, the implications of our study, which indicate the need for focusing on patient clinical features and neuroimaging, were very clearly demonstrated, even with the limitations. A surprising result was that ILR monitoring detected AF in 34.1% of the study patients within less than 60 days. Thus, we thought that it is important to intensive monitoring of AF cause in patients with embolic stroke of undetermined source. Also, we did not investigate the potential effects of cardiac factors (such as a left atrial enlargement, elevated cardiac markers, etc.); however, this is beyond the scope of the current analysis. We did not validate other types of ILR devices, although a small prospective study showed that Medtronic cardiac monitoring devices effectively detected covert AF in patients with ESUS. It is not yet clear whether other types of ILR are effective in detecting covert AF in patients with ESUS, and prospective large-scale, multicenter clinical trials are needed to investigate this matter. More specific and uniform criteria are also needed for the classification of patients with ESUS. Our results are also limited by the lack of assessment of patients who had a devastating stroke, but the number of patients in this category was small.

In conclusion, our study showed that AF was more frequently detected by an ILR than in previous reports examining recent ESUS. Higher covert AF detection rates within the ESUS patients were most often associated with higher NIHSS and whole-territorial infarction patterns in neuroimaging.

## Data Availability Statement

The original contributions presented in the study are included in the article/Supplementary Material, further inquiries can be directed to the corresponding author/s.

## Ethics Statement

The studies involving human participants were reviewed and approved by Jeju National University Hospital, Institutional Review Board. Written informed consent for participation was not required for this study in accordance with the national legislation and the institutional requirements.

## Author Contributions

J-GK, C-HK, HK, and JC contributed to the article by participating in the concept and design, acquisition of data, and critical revision of the manuscript for intellectual content. J-GK and JC contributed in analysis, interpretation of data, and drafting of the manuscript. All authors contributed to data interpretation, writing, editing, and revising the final manuscript, and the production of the final version of this manuscript.

## Funding

This work was supported by a research grant from Jeju National University Hospital in 2020.

## Conflict of Interest

The authors declare that the research was conducted in the absence of any commercial or financial relationships that could be construed as a potential conflict of interest.

## Publisher's Note

All claims expressed in this article are solely those of the authors and do not necessarily represent those of their affiliated organizations, or those of the publisher, the editors and the reviewers. Any product that may be evaluated in this article, or claim that may be made by its manufacturer, is not guaranteed or endorsed by the publisher.
