# Supplementary material for: Impact of Neuroimaging Patterns for the Detection of Atrial Fibrillation by Implantable Loop Recorders in Patients With Embolic Stroke of Undetermined Source
Source: Front Neurol. 2022 Jun 13;13:905998. doi: 10.3389/fneur.2022.905998 (PMC9234145; doi:10.3389/fneur.2022.905998)
Supplement: Supplementary file 1 [file Table_1.DOCX]

| Supplementary data 1: Characteristics of individual patients characteristics who were diagnosed with atrial fibrillation during the study | | | | | | | | | | | | | | | | |
| --- | --- | --- | --- | --- | --- | --- | --- | --- | --- | --- | --- | --- | --- | --- | --- | --- |
|  | Age | Sex | BMI | Initial NIHSS | CHA₂DS₂-VASc Score | Time of diagnosis | A-fib duration (s) | Max Rate (bpm) | Max Rate (ms) | Median rate (BPM) | Median rate (ms) | Pattern | Vascular territory | Time to transthoracic echocardiography | Time to Holter monitoring | Time to the first detection of atrial fibrillation |
| 1 | 54 | Male | 22.84 | 13 | 0 | 2:55 | 16 | 154 | 390 | 154 | 390 | Whole-territorial infarction | MCA | 8 | 1 | 58 |
| 2 | 77 | Male | 24.67 | 5 | 3 | 10:00 | 179 | 375 | 160 | 375 | 160 | Whole-territorial infarction | MCA | 14 | 3 | 295 |
| 3 | 43 | Male | 23.59 | 16 | 0 | 15:37 | 120 | 146 | 410 | 143 | 420 | Whole-territorial infarction | MCA | 3 | 5 | 39 |
| 4 | 71 | Male | 20.61 | 6 | 2 | 23:33 | 103560 | 200 | 300 | 143 | 420 | Whole-territorial infarction | MCA | 17 | 3 | 45 |
| 5 | 59 | Male | 24.20 | 13 | 2 | 9:10 | 120 | 167 | 350 | 115 | 520 | Whole-territorial infarction | MCA | 3 | 3 | 52 |
| 6 | 64 | Male | 22.84 | 0 | 0 | 10:08 | 9000 | 150 | 400 | 91 | 660 | Whole-territorial infarction | MCA | 2 | 3 | 174 |
| 7 | 82 | Female | 20.95 | 7 | 2 | 3:29 | 99360 | 171 | 350 | 125 | 480 | Multiple-territorial | BA | 3 | 3 | 72 |
| 8 | 58 | Female | 26.07 | 1 | 1 | 10:01 | 113 | 176 | 340 | 158 | 380 | Scattered | MCA | 2 | 2 | 49 |
| 9 | 59 | Male | 27.08 | 20 | 0 | 6:02 | 42 | 154 | 390 | 154 | 390 | Whole-territorial infarction | MCA | 2 | 2 | 53 |
| 10 | 62 | Male | 27.31 | 0 | 0 | 17:11 | 125040 | 188 | 320 | 75 | 800 | Multiple-territorial | ICA | 5 | 5 | 67 |
| 11 | 66 | Male | 20.76 | 16 | 1 | 7:00 | 27 | 222 | 270 | 207 | 290 | Whole-territorial infarction | MCA | 11 | 6 | 44 |
| 12 | 88 | Female | 27.49 | 16 | 5 | 15:00 | 2400 | 122 | 490 | 91 | 660 | Whole-territorial infarction | MCA | 2 | 2 | 45 |
| 13 | 73 | Female | 26.65 | 6 | 3 | 9:50 | 120 | 188 | 320 | 105 | 570 | Lobar | MCA | 3 | 3 | 44 |
| 14 | 64 | Male | 27.22 | 2 | 0 | 9:36 | 480 | 214 | 280 | 143 | 420 | Lobar | MCA | 77 | 3 | 60 |
| BMI: Body mass index, NIHSS: National Institutes of Health Stroke Scale, A-fib: atrial fibrillation, BPM: beats per minute, MCA: middle cerebral artery, BA: basilar artery, ICA: internal carotid artery | | | | | | | | | | | | | | | | |
